# Neonatal, Infant, and Under Age Five Vaccine Doses Routinely Given in Developed Nations and Their Association With Mortality Rates

**DOI:** 10.7759/cureus.42194

**Published:** 2023-07-20

**Authors:** Neil Z Miller, Gary S Goldman

**Affiliations:** 1 Medical Research, Institute of Medical and Scientific Inquiry, Santa Fe, USA; 2 Research, Independent Computer Scientist, Bogue Chitto, USA

**Keywords:** infant mortality rate (imr), hepatitis b vaccine, sudden infant death syndrome (sids), neonatal mortality, linear regression, non-specific effects, preterm, under 5 mortality, vaccination, immunization

## Abstract

Introduction

In 2011, using 2009 data, we published a study demonstrating that among the most highly developed nations, those requiring the most vaccine doses for their infants tended to have the least favorable infant mortality rates (*r *= 0.70, *p *< .0001). Twelve years later, we replicated our original study using 2019 data. Linear regression analysis corroborated the positive trend reported in our initial paper (*r *= 0.45; *p *< .002). Herein, we broaden our analyses to consider the effect of vaccines on neonatal and under age five mortality rates.

Objective

We performed several investigations to explore potential relationships between the number of early childhood vaccine doses required by nations and their neonatal, infant, and under age five mortality rates.

Methods

In this ecological study, we conducted linear regression analyses of neonatal, infant, and under age five vaccine doses required by nations and their neonatal, infant, and under age five mortality rates. All analyses were based on 2019 and 2021 data. We also stratified nations by the number of neonatal vaccine doses required and conducted a one-way ANOVA test and a post hoc Tukey-Kramer test to determine if there were statistically significant differences in the group mean neonatal, infant, and under age five mortality rates of nations that administered zero, one, or two neonatal vaccine doses.

Results

Linear regression analyses of neonatal vaccine doses required by nations in our 2021 dataset yielded statistically significant positive correlations to rates of neonatal mortality (*r *= 0.34, *p* = .017), infant mortality (*r *= 0.46, *p* = .0008), and under age five mortality (*r *= 0.48, *p* = .0004). Similar results were reported using 2019 data.

Utilizing 2021 data, a post hoc Tukey-Kramer test indicated a statistically significant pairwise difference between the mean neonatal mortality rates, mean infant mortality rates, and mean under age five mortality rates of nations requiring zero vs. two neonatal vaccine doses. There was a statistically significant difference of 1.28 deaths per 1000 live births (*p* < .002) between the mean infant mortality rates among nations that did not give their neonates any vaccine doses and those that required two vaccine doses.

Using 2019 and 2021 data, 17 of 18 analyses (12 bivariate linear regressions and six ANOVA and Tukey-Kramer tests) achieved statistical significance and corroborated the findings reported in our original study of a positive association between the number of vaccine doses required by developed nations and their infant mortality rates.

Conclusions

There are statistically significant positive correlations between mortality rates of developed nations and the number of early childhood vaccine doses that are routinely given. Further investigations of the hypotheses generated by this study are recommended to confirm that current vaccination schedules are achieving their intended objectives.

## Introduction

According to global health experts, "Few measures in public health can compare with the impact of vaccines. Vaccinations have reduced disease, disability, and death from a variety of infectious diseases" [1]. The US Centers for Disease Control and Prevention (CDC) estimates that four million deaths are prevented by childhood vaccination every year [2]. The World Health Organization (WHO) has calculated that an additional 1.5 million deaths could be averted if global vaccination coverage rates are improved [3].

Although the protective benefits of vaccination are well-established in the medical literature, some studies have challenged the idea that when more vaccines are administered, it always results in fewer deaths. For example, Aaby et al. [4] discovered that in some low-income nations vaccines have non-specific effects (NSEs) that can increase or decrease mortality from infectious diseases not targeted by the vaccine. Mogensen et al. [5] found that all-cause infant mortality in Guinea-Bissau increased twofold after diphtheria-tetanus-pertussis (DTP) and oral polio vaccines were introduced (hazard ratio, HR = 2.12). Survival rates in infants who received DTP only (without an oral polio vaccine) as compared to non-DTP vaccinated children were significantly worse (HR = 10.0). Differences in background factors did not explain the effect.

Fisker and Thysen [6] found that the sequence of administered vaccines affects all-cause mortality. Girls who received a pentavalent vaccine (DTP-Haemophilus influenzae type b-hepatitis B) *after* receiving a measles vaccine were five times more likely to die from all causes within six months of follow-up when compared with girls who received vaccines in the recommended sequence (HR = 5.10). The authors rendered a cautionary statement: "It is assumed that providing missing vaccine doses will always leave the child better off than not providing them. This may be wrong."

There are other ways to investigate whether providing more versus fewer vaccine doses is always ideal. In 2011, using 2009 data, we observed that several nations had better infant mortality rates (IMRs) than the US, yet they all required fewer vaccine doses for their infants than the US. Thus, we conducted a study [7] to explore a potential relationship between the number of vaccine doses that these nations required of their infants and their IMRs. Linear regression analysis yielded a coefficient of determination, *r*^2^ = 0.49 (*r* = 0.70; *p* < .0001). Among the 30 nations with the best IMRs, those requiring the most vaccine doses for their infants tended to have the least favorable IMRs. This positive correlation challenges the conventional scientific consensus that more vaccine doses always equate with fewer deaths.

Twelve years later, we published a response [8] to several claims made by critics of our paper. In addition, we replicated our original study using 2019 data. Linear regression analysis yielded a coefficient of determination, *r*^2^ = 0.20 (*r* = 0.45; *p* < .002), corroborating the positive trend reported in our initial paper.

In most nations, more than half of all infant deaths occur during the neonatal period (the first 28 days of life). Therefore, in this present study, we broaden our analyses to explore potential relationships between the number of neonatal vaccine doses required by nations and their neonatal mortality rates (NMRs), IMRs, and under age five mortality rates (U5MRs). We also investigate further relationships between infant vaccine doses required by nations and their IMRs and U5MRs. Finally, we investigate a relationship between the number of vaccine doses that nations required during the first five years of life and their U5MRs.

## Materials and methods

The data discussed in this paper supplements analyses conducted in our previous papers [7,8]. In this present ecological study, we used simple linear regression (i.e., bivariate regression) between the number of vaccine doses (the independent variable) that nations routinely require and these nations' reported mortality rates (the dependent variable). Thus, we conducted twelve different linear regression analyses (six each for 2019 and 2021) to investigate potential correlations between the number of vaccine doses that the nations in our datasets required of their neonates, infants, and under age five children and these nations' neonatal, infant, and under age five mortality rates. For example, by conducting separate bivariate, linear regressions between infant vaccine doses (given <1 year of age) and nations' IMRs and then U5MRs, we sought to explore the short-term influence, and then the more longitudinal impact, of routinely administered infant vaccine doses.

While there are multiple independent variables that influence a nation's mortality rate, our study design focused solely on the influence of the number of vaccine doses required by developed nations with relatively homogenous socioeconomic factors and high vaccination rates. Therefore, no multiple regression analyses were conducted. This simplification avoided the increased (a) complexity of including additional variables in the analysis, (b) risk of confounding, (c) sample size requirements to achieve statistical power, and (b) difficulty interpreting the results and drawing meaningful conclusions.

Additionally, we conducted six different analyses of variance (one-way ANOVA) tests (three each for 2019 and 2021) to determine if there were statistically significant differences in the group mean neonatal, infant, and under age five mortality rates of the nations in our datasets after stratifying these mortality rates by the number of neonatal doses routinely given (zero, one, or two). A Tukey-Kramer test was utilized to identify any statistically significant pairwise difference between the group means. Since, in some cases, the distribution of the mortality rates in any given group may not satisfy normality, a Kruskal-Wallis nonparametric test that ranks the mortality rates was also performed to confirm that a statistically significant pairwise difference in mortality rate rankings exists among the same pairwise groups reported by the Tukey-Kramer test.

To remain consistent with analyses conducted in our previous papers, the 2019 and 2021 datasets include the US, a nation that required the most vaccines for their infants, and all nations with better IMRs than the US. Nations reporting six or fewer infant deaths (Andorra, Antigua and Barbuda, Monaco, and San Marino) were omitted from the analyses due to IMR instability caused by excessively wide confidence intervals. Thus, our present analyses of 2019 and 2021 data utilize the top-ranking 44 and 50 nations, respectively, reporting the best IMRs.

Note: This epidemiological convention of removing small nations with few infant deaths and IMR instability is recommended by the CDC and other health agencies. This was thoroughly discussed in our previous paper [8]. Although there were no appreciable differences in the reported results when we added these nations back into our datasets and reanalyzed the data, to avoid the perception of bias we provide supporting tables and results in the appendix.

IMRs were amassed from the UNICEF data warehouse [9]. NMRs and U5MRs were retrieved from UNICEF global databases [10]. Immunization schedules and the number of neonatal, infant, and under age five vaccine doses required by each nation were collected from the World Health Organization, the European Centre for Disease Prevention and Control, and national governments [11,12]. Combination vaccines administered via a single injection, such as MMR (for measles, mumps, and rubella), were counted by the number of vaccine doses they actually contain.

The linear regression calculator to determine the slope of the best-fit line, the correlation coefficient (*r*) and corresponding *p*-value, and the ANOVA test (with Tukey-Kramer post analysis), are available online at: https://www.statskingdom.com. Statistical significance is considered to have been achieved when *p* < .05. The mean mortality rates (with 95% confidence intervals) stratified by number of neonatal vaccine doses were calculated and graphically depicted using the online ANOVA summary software available at https://acetabulum.dk/anova.html. Software used to produce the scatter plot (with best-fit line and 95% confidence band) is freely available online at https://acetabulum.dk/cor.test.html.

We did not analyze 2020 data because changes to mortality rates and immunization schedules from the previous year were negligible. Mortality rates for 2021 were the most recent available. Similar analyses were not performed on our original 2011 study (using 2009 data) that consisted of the top-ranking 30 nations due to low or insufficient statistical power caused by small sample size, especially after further stratifying nations into subgroups that specify zero, one, or two neonatal vaccine doses.

## Results

Linear regression analyses, 2019 and 2021

**Figure 1:**
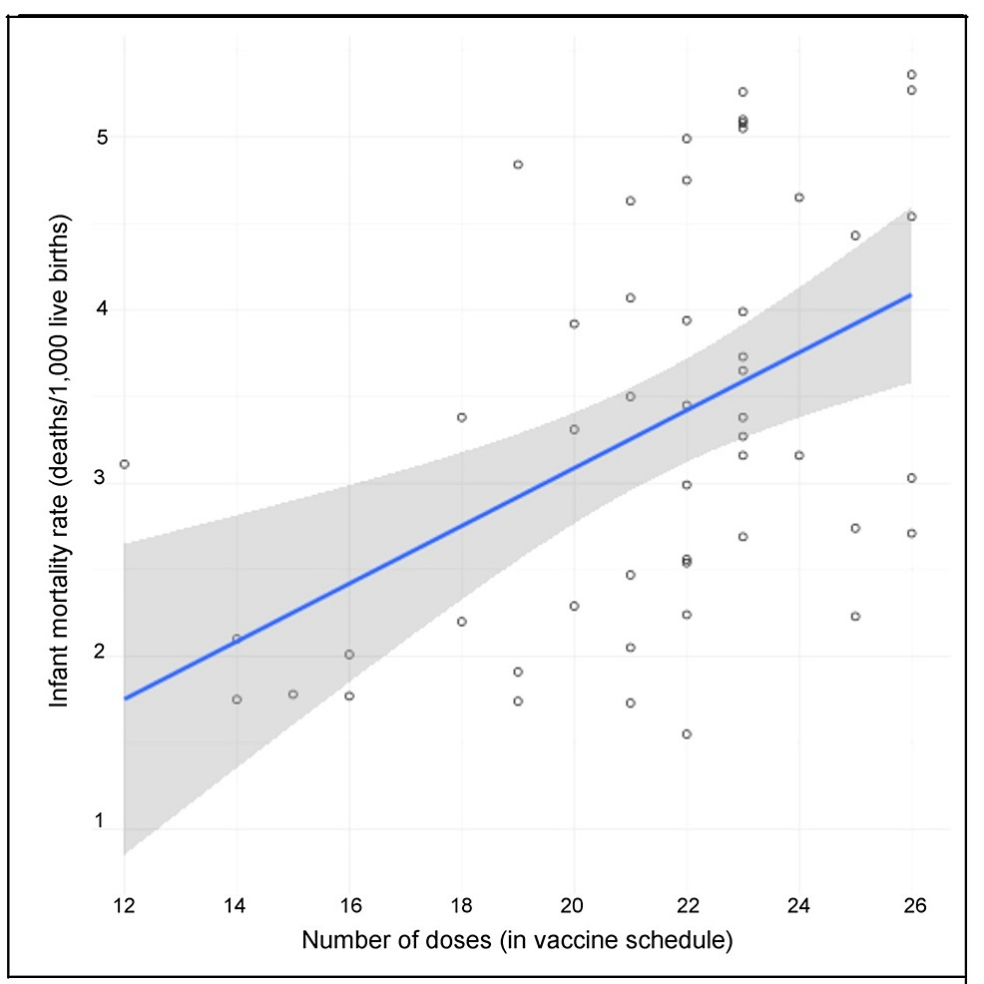
Scatter plot of infant mortality rates versus number of infant vaccine doses, with best-fit line and 95% confidence band (shaded region), 2021 (n = 50), r = 0.47 (p = .0005)

Based on the 2019 data given in Table 1 and 2021 data given in Table 2, the 12 (i.e., six for each year) applicable combinations of linear regression analyses of NMR, IMR, and U5MR vs. the number of neonatal, infant, and under age five vaccine doses specified by each nation, all yield statistically significant positive correlations as shown in Table 3. For our 2021 linear regression analysis of infant vaccine doses and IMR, a scatter plot with a best-fit line is provided in Figure 1.

**Table 1:** Nations stratified into groups of 0, 1, and 2 neonatal vaccine doses routinely given, with the number of vaccine doses and mortality rates for each childhood period, 2019 Dataset: 44 nations (The US and all nations with better IMRs) NMRs: Neonatal Mortality Rates; IMRs: Infant Mortality Rates; U5MRs: Under Age 5 Mortality Rates; d: days; y: years ^a^Nations are listed in the order of their IMRs ^b^Nations only giving BCG vaccine (for tuberculosis)

Groups stratified by number of neonatal doses	Nations	Doses <1y	Doses <5y	NMRs <28d	IMRs^a^ <1y	U5MRs <5y
Group #1: Nations giving 0 neonatal doses, n = 22 (50%)	Iceland	14	26	1.38	1.59	2.69
	Slovenia	17	26	1.35	1.82	2.30
	Japan	19	33	0.85	1.85	2.47
	Norway	16	26	1.39	1.88	2.35
	Finland	15	29	1.44	1.91	2.27
	Sweden	16	25	1.44	2.18	2.63
	Cyprus	20	29	1.62	2.29	2.80
	Luxembourg	22	36	1.69	2.30	2.81
	Czechia	18	24	1.58	2.43	3.02
	Italy	25	28	1.74	2.58	2.99
	Spain	22	31	1.84	2.71	3.17
	Austria	26	28	2.26	2.94	3.58
	Germany	22	38	2.27	3.16	3.73
	Denmark	12	24	2.63	3.24	3.78
	France	22	29	2.59	3.41	4.35
	Belgium	23	34	2.40	3.42	4.11
	Netherlands	21	29	2.65	3.53	4.05
	Switzerland	18	29	2.79	3.55	3.98
	Greece	26	38	2.42	3.65	4.03
	U.K.	23	38	2.84	3.68	4.32
	New Zealand	23	36	2.65	4.05	4.93
	Slovakia	21	24	2.91	4.77	5.82
Group #2: Nations giving 1 neonatal dose (either Hep B or BCG), n = 12 (27%)	Estonia^b^	22	30	1.02	1.77	2.29
	Montenegro	19	27	1.19	2.13	2.70
	Belarus	18	24	1.09	2.36	3.13
	Ireland^b^	26	37	2.17	2.73	3.31
	Portugal	20	30	1.89	2.84	3.44
	Israel	23	34	1.85	2.96	3.54
	Australia	23	39	2.36	3.16	3.75
	Hungary^b^	18	29	2.18	3.42	4.12
	Latvia^b^	24	36	2.22	3.53	3.98
	Croatia^b^	20	30	2.92	3.94	4.72
	Canada	25	41	3.44	4.44	5.18
	U.S.	26	38	3.53	5.52	6.44
Group #3: Nations giving 2 neonatal doses (both Hep B and BCG), n = 10 (23%)	Singapore	21	33	0.89	1.97	2.40
	South Korea	21	35	1.49	2.68	3.12
	Lithuania	26	36	2.08	2.95	3.76
	Poland	22	31	2.74	3.81	4.42
	Cuba	23	33	2.34	4.17	5.22
	Russia	21	29	2.64	4.70	5.84
	Serbia	22	31	3.62	4.93	5.68
	Bosnia & H.	19	28	4.31	5.06	5.88
	Qatar	26	45	3.62	5.25	6.06
	Bulgaria	23	32	3.26	5.49	6.70

**Table 2:** Nations stratified into groups of 0, 1, and 2 neonatal vaccine doses routinely given, with number of vaccine doses and mortality rates for each childhood period, 2021 Dataset = 50 nations (The US and all nations with better IMRs) NMRs: Neonatal Mortality Rates; IMRs: Infant Mortality Rates; U5MRs: Under Age 5 Mortality Rates; d: days; y: years ^a^Nations are listed in the order of their IMRs ^b^Nations giving only BCG vaccine (for tuberculosis)

Groups stratified by number of neonatal vaccine doses	Nations	Doses <1y	Doses <5y	NMRs <28d	IMRs^a^ <1y	U5MRs <5y
Group #1: Nations giving 0 neonatal vaccine doses, n = 24 (48%)	Japan	19	33	0.81	1.74	2.30
	Slovenia	14	24	1.28	1.75	2.17
	Norway	16	26	1.29	1.77	2.17
	Finland	15	29	1.27	1.78	2.17
	Sweden	16	25	1.35	2.01	2.46
	Iceland	14	28	1.36	2.10	2.64
	Czechia	18	24	1.44	2.20	2.75
	Italy	25	28	1.47	2.23	2.61
	Luxembourg	22	37	1.65	2.24	2.73
	Cyprus	20	29	1.61	2.29	2.79
	Spain	22	31	1.78	2.56	3.05
	Ireland	25	36	2.06	2.74	3.15
	Austria	22	26	2.34	2.99	3.66
	Germany	26	31	2.19	3.03	3.57
	Denmark	12	24	2.48	3.11	3.58
	Greece	23	38	2.25	3.27	3.71
	Belgium	23	34	2.43	3.38	4.10
	Switzerland	18	29	2.70	3.38	3.83
	France	22	29	2.53	3.45	4.35
	Netherlands	21	29	2.68	3.50	4.05
	U.K.	23	38	2.80	3.65	4.19
	New Zealand	22	35	2.54	3.94	4.73
	Slovakia	21	27	2.80	4.63	5.63
	Malta	23	38	3.87	5.08	5.83
Group #2: Nations giving 1 neonatal vaccine dose (either Hep B or BCG), n = 12 (24%)	Estonia^b^	22	30	0.88	1.55	2.00
	Montenegro	19	27	0.96	1.91	2.26
	Belarus	21	29	0.87	2.05	2.72
	Portugal	22	33	1.71	2.54	3.10
	Israel	23	34	1.73	2.69	3.36
	Australia	23	40	2.37	3.16	3.71
	Latvia^b^	24	36	2.05	3.16	3.68
	Hungary^b^	20	32	2.07	3.31	4.00
	Croatia^b^	20	30	2.78	3.92	4.64
	Canada	25	41	3.41	4.43	5.04
	Uruguay^b^	22	38	4.00	4.99	5.82
	U.S.	26	38	3.27	5.36	6.24
Group #3: Nations giving 2 neonatal vaccine doses (both Hep B and BCG), n = 14 (28%)	Singapore	21	33	0.74	1.73	2.09
	South Korea	21	35	1.38	2.47	2.89
	Lithuania	26	36	1.91	2.71	3.31
	Poland	23	32	2.76	3.73	4.35
	Cuba	23	33	2.38	3.99	5.00
	Russia	21	29	2.04	4.07	5.05
	Qatar	26	45	3.30	4.54	5.31
	North Macedonia	24	33	3.41	4.65	5.31
	Serbia	22	31	3.55	4.75	5.49
	Bosnia & H.	19	28	4.10	4.84	5.60
	China	23	33	3.19	5.05	6.93
	Maldives	23	29	4.06	5.10	5.96
	Romania	23	26	3.23	5.26	6.43
	Bulgaria	26	35	3.01	5.27	6.32

**Table 3:** Correlation coefficients (r) reported by linear regression analysis between the number of vaccine doses given during each childhood period and each mortality rate, 2019 and 2021 ^a^These values are not applicable (N/A) since many of the vaccine doses are given after the cutoff period.

	2019 correlation coefficients (r)			2021 correlation coefficients (r)		
	Neonatal mortality rates	Infant mortality rates	Under age 5 mortality rates	Neonatal mortality rates	Infant mortality rates	Under age 5 mortality rates
Neonatal doses	0.31 (p=.04)	0.45 (p=.002)	0.46 (p=.002)	0.34 (p=.017)	0.46 (p=.0008)	0.48 (p=.0004)
Infant doses	N/A^a^	0.45 (p=.002)	0.42 (p=.005)	N/A^a^	0.47 (p=.0005)	0.46 (p=.0007)
Under age 5 doses	N/A^a^	N/A^a^	0.32 (p=.034)	N/A^a^	N/A^a^	0.30 (p=.031)

ANOVA and post hoc Tukey-Kramer tests, 2019 and 2021

Based on an ANOVA test utilizing 2019 mortality rates, the mean NMRs corresponding to groups of nations requiring zero, one, and two neonatal vaccine doses demonstrated no statistically significant difference. ANOVA did, however, report a statistically significant difference between the group means of IMRs and U5MRs. A post hoc Tukey-Kramer test indicated a statistically significant pairwise difference specifically between each of the group mean IMRs (*p* = .006) and U5MRs (*p* = .004) of nations requiring zero versus two neonatal vaccine doses (Table 4).

**Table 4:** 2019 group mean mortality rates of nations requiring zero, one, or two neonatal vaccine doses, plus ANOVA and Tukey-Kramer p-values of differences between the group means ^a^Of the 44 nations in the dataset, 22 (50%), 12 (27.3%), and 10 (22.7%) required their newborns receive zero, one, and two neonatal vaccine doses, respectively. ^b^Post hoc Tukey-Kramer tests for 2019 demonstrated a statistically significant (*p* < .05) pairwise difference between mean infant mortality rates (IMRs) and mean under age 5 mortality rates (U5MRs) of nations requiring zero versus two neonatal doses. While the difference between the 2019 mean neonatal mortality rates (NMRs) of nations requiring zero versus two neonatal doses was not statistically significant at the 95% confidence level, it was statistically significant at the 91% confidence level.

	2019 number of neonatal vaccine doses^a^			p-value corresponding to...	
	Zero	One	Two	ANOVA	Tukey-Kramer^b^
Mean NMR	2.03	2.16	2.70	.093	.089
Mean IMR	2.86	3.23	4.10	.008	.006
Mean U5MR	3.46	3.88	4.91	.006	.004

Based on an ANOVA test utilizing 2021 mortality rates, the mean NMRs, IMRs, and U5MRs corresponding to groups of nations requiring zero, one, and two neonatal vaccine doses, each demonstrated a statistically significant difference between the group means. A post hoc Tukey-Kramer test indicated a statistically significant pairwise difference specifically between each of the group mean NMRs (*p* = .013), IMRs (*p* = .002), and U5MRs (*p* = .001) of nations requiring zero vs. two neonatal vaccine doses (Table 5).

**Table 5:** 2021 group mean mortality rates of nations requiring zero, one, or two neonatal vaccine doses, plus ANOVA and Tukey-Kramer p-values of differences between the group means ^a^Of the 50 nations in the dataset, 24 (48%), 12 (24%), and 14 (28%) required their newborns receive zero, one, and two neonatal vaccine doses, respectively. ^b^All post hoc Tukey-Kramer tests for 2021 demonstrated a statistically significant (*p* < .05) pairwise difference between the mean mortality rates of nations requiring zero versus two neonatal doses. NMR: neonatal mortality rate; IMR: infant mortality rate: U5MR: under age five mortality rate

	2021 number of neonatal vaccine doses^a^			p-value corresponding to...	
	Zero	One	Two	ANOVA	Tukey-Kramer^b^
Mean NMR	1.96	2.18	2.79	.017	.013
Mean IMR	2.87	3.26	4.15	.003	.002
Mean U5MR	3.43	3.88	5.00	.002	.001

**Figure 2:**
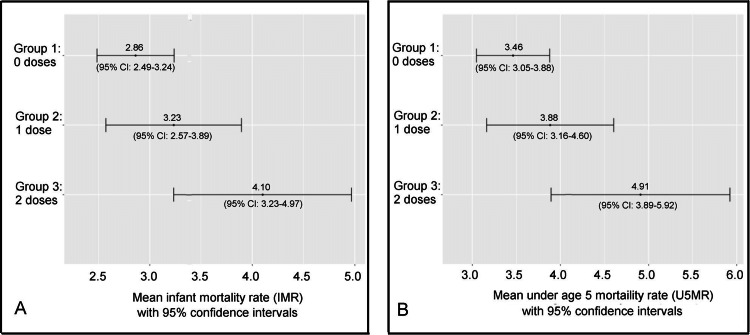
2019 group mean infant and under age 5 mortality rates (with 95% confidence intervals) for nations stratified by the number of neonatal vaccine doses given (A) Mean infant mortality rates with 95% confidence intervals (B) Mean under age 5 mortality rates with 95% confidence intervals

**Figure 3:**
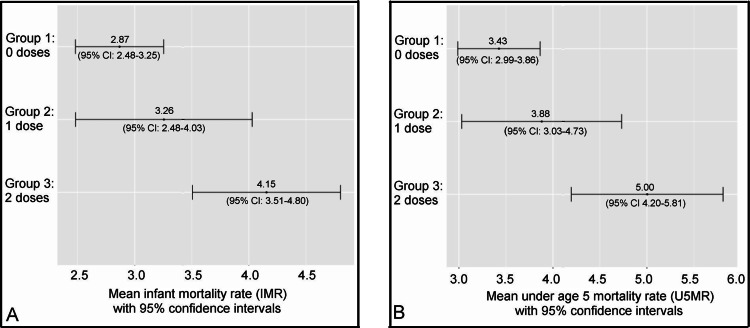
2021 group mean infant and under age 5 mortality rates (with 95% confidence intervals) for nations stratified by the number of neonatal vaccine doses given (A) Mean infant mortality rates with 95% confidence intervals (B) Mean under age 5 mortality rates with 95% confidence intervals

Confidence intervals of mean infant mortality rates and under age five mortality rates for nations stratified by the number of neonatal vaccine doses given are shown in Figure 2 and Figure 3, for the years 2019 and 2021, respectively.

Reporting of the slopes of the best-fit lines

The slopes of the best-fit lines (with 95% confidence intervals) for 2019 and 2021, derived from linear regression analysis of mortality rates versus number of vaccine doses for each childhood period, are shown in Table 6.

**Table 6:** Slopes of the best-fit line from each linear regression analysis of mortality rate versus the number of vaccine doses during each childhood period, 2019 and 2021 ^a^Slope represents the rate of change in deaths/1000 live births per vaccine dose. The correlation coefficient and corresponding *p*-values are given in Table 3. NMR: Neonatal Mortality Rate; IMR: Infant Mortality Rate; U5MR: Under Age 5 Mortality Rate

Linear regression analysis	2019 Slope^a^ (95% CI)	2021 Slope^a^ (95% CI)
NMR vs. neonatal vaccine doses	0.309 (0.0140 – 0.604)	0.359 (0.0662 – 0.651)
IMR vs. infant vaccine doses	0.141 (0.0533 – 0.229)	0.167 (0.0766 – 0.257)
U5MR vs. under age 5 vaccine doses	0.077 (0.0061 – 0.149)	0.087 (0.0080 – 0.165)

## Discussion

In 2011, using 2009 data [7], our linear regression analysis of IMR vs. the number of infant vaccine doses required by each nation in our dataset yielded a statistically significant positive correlation of *r* = 0.70 (*p* < .0001), indicating that among the most highly developed nations, those requiring the most vaccine doses for their infants tend to have the worst IMRs. Twelve years later, using 2019 data [8], we found a statistically significant positive correlation of *r* = 0.45 (*p* = .002), which corroborated the positive trend reported in our initial study. In this current paper, we found several additional statistically significant correlations between NMRs, IMRs, and U5MRs of developed nations and the number of early childhood vaccine doses routinely given to children during each childhood period.

Neonatal vaccines

Many nations require hepatitis B and/or BCG (bacille Calmette-Guerin) vaccines (for tuberculosis) to be administered to newborns as soon as possible following birth. In 1991, the CDC initially recommended the hepatitis B vaccine for all US infants "to eliminate hepatitis B virus transmission" [13] although just 0.6% of hepatitis B cases occurred in children less than 15 years of age [14]. A few years later, the CDC and World Health Organization recommended that the first dose of the hepatitis B vaccine be given within 24 hours after birth to prevent perinatal transmission of the virus [13,15]. The BCG vaccine is given to newborns in many nations to prevent childhood tuberculous meningitis and miliary disease [16]. This vaccine is usually not required for newborns in nations with a low risk of infection.

Using 2019 and 2021 datasets, linear regression analysis yielded statistically significant positive correlations between the number of neonatal vaccine doses required by nations and NMR (*r* = 0.31, 0.34), IMR (*r* = 0.45, 0.46), and U5MR (*r* =0.46, 0.48). Additionally, statistically significant differences were reported between the 2019 group mean IMRs and U5MRs, and the 2021 group mean NMRs, IMRs, and U5MRs, of nations requiring zero vs. two neonatal vaccine doses.

Although fatalities after hepatitis B vaccination have been documented, evidence regarding a causative effect remains inconclusive. Approximately 60 deaths in all age groups and 30 deaths among infants following hepatitis B vaccination are reported annually to the US Vaccine Adverse Event Reporting System (VAERS) [17]. However, these figures are likely biased low due to underreporting since parents, as well as physicians and medical professionals, are taught to accept vaccines as "safe and effective" and reject the possibility that a death could be vaccine-related. Coroners are unlikely to conduct thorough postmortem examinations necessary to reveal the cause of death as related to hepatitis B vaccination. In fact, a report prepared by Harvard Pilgrim Health Care [18] for the U.S. Department of Health and Human Services (HHS) found that "fewer than 1% of vaccine adverse events are reported."

In a 2012 study, we found that a disproportionate number of hospitalizations and deaths were associated with the neonatal dose of the hepatitis B vaccine [19]. Other studies provide additional evidence of correlations between hepatitis B vaccination and serious adverse reactions. For example, Gallagher and Goodman [20] found that boys who received a hepatitis B vaccine during the neonatal period were three times more likely than non-vaccinated neonates to later be diagnosed with autism (OR = 3.0). Top epidemiologists working for the CDC [21] found that children who received a thimerosal-containing hepatitis B vaccine during the neonatal period "had a 7.6 times higher risk of autism diagnosis (95% CI of 1.8 to 31.5) than their unexposed peers." They were also significantly more likely to develop neurodevelopmental disorders, and sleep and speech disorders. If premature neonates had not been excluded from this study, rates of deleterious health outcomes might have been even higher.

Hernán et al. [22] found that patients with multiple sclerosis were three times more likely to have been vaccinated against hepatitis B within three years before the date of first symptoms when compared to controls who were not vaccinated (OR = 3.1). Geier and Geier [23] found that recipients of hepatitis B vaccination were five times more likely to develop multiple sclerosis when compared to controls (OR = 5.2), and had higher rates of arthritis, optic neuritis, lupus, vasculitis, and thrombocytopenia.

A CDC-sponsored study by Eriksen et al. [24] evaluated 90 neonatal deaths and found "no significant difference in the proportion of hepatitis B-vaccinated and unvaccinated neonates dying of unexpected causes." However, among the vaccinated newborns, 36% of the unexpected deaths were from Sudden Infant Death Syndrome (SIDS); there were no SIDS cases in the unvaccinated group. A major weakness in the study methodology was a failure to match the unvaccinated neonates to vaccinated neonates for birth weight. The median birth weight of the vaccinated newborns (3100 grams) was significantly higher (*p* < 0.01) than that of the unvaccinated newborns (1358 grams). Low birth weight is a strong predictor of neonatal mortality and was the primary cause of neonatal mortality in this study. Apgar scores were also significantly better for the hepatitis B-vaccinated versus unvaccinated neonates (*p* < 0.01). These confounders invalidate the study results and conclusions.

An FDA-sponsored study by Niu et al. [25] investigated 18 neonatal deaths that occurred shortly after hepatitis B vaccination. Of the 18 deaths, 12 (67%) were recorded by the medical examiner as due to SIDS. The authors state that "coincidental SIDS deaths are expected following any infant vaccination." The remaining deaths were also recorded as due to causes other than hepatitis B vaccination. The authors conclude that "hepatitis B vaccination is not causing a clear increase in unexplained neonatal or infant deaths."

In China, 17 infant deaths following hepatitis B vaccination occurred in a single month [26]. Several of the deaths were in neonates and happened within hours post-vaccination. Media reports about the deaths caused parental confidence in the vaccine, and hepatitis B vaccination rates, to significantly decline. An investigation by the China Food and Drug Administration concluded that none of the infant deaths were caused by the vaccine; all of the deaths were determined to be "coincidental events."

Although some study authors have concluded that sudden or unexpected deaths following the neonatal dose of the hepatitis B vaccine are merely coincidental, it is possible and even likely that some of these deaths were actually precipitated by the vaccine. The neonatal dose of the hepatitis B vaccine, administered at a time when the immune system is immature and the neonate has low birth weight, may increase vulnerability to serious adverse reactions and deaths that ultimately contribute to higher NMRs, IMRs, and U5MRs.

Non-specific effects (NSEs) of vaccines

Vaccines have NSEs (or heterologous effects) on morbidity and mortality. Numerous studies indicate that live vaccines such as BCG, measles, and oral polio reduce overall mortality in low-income nations [27-29]. In contrast, non-live vaccines such as hepatitis B, DTP, Hib (Haemophilus influenzae type b), and inactivated polio increase susceptibility to non-targeted infections. The deleterious NSEs are often more pronounced than the beneficial targeted effects, thus all-cause mortality is significantly higher. In a study by Garly et al. [30], hepatitis B-vaccinated infants had higher overall mortality than infants who had not received the hepatitis B vaccine (mortality rate = 1.81; 95% CI: 1.19 to 2.75). The deleterious effect was even stronger for girls (mortality rate = 2.27; 95% CI: 1.31 to 3.94).

Although studies conducted in low-income nations indicate that the BCG vaccine has beneficial NSEs, a large randomized trial in Denmark [31], a high-income nation, found that BCG vaccination at birth did not reduce the risk of hospitalization for acquired somatic morbidity (all-cause hospitalizations excluding injuries). This finding of no differences between vaccinated and unvaccinated cohorts remained consistent in males and females, across study sites, and in children born at term and prematurely. Two large randomized trials in India [32] found that BCG vaccination at birth had no effect on neonatal mortality. There was no reduction in mortality from either the targeted infection or non-targeted infections.

Since tuberculosis is rare in developed nations (about 87% of new cases occur in just 30 low- to middle-income nations) [33], the BCG vaccine given shortly after birth is unlikely to have an appreciable positive effect on reducing deaths from the disease. Instead, its effect may be neutral like the findings in the Denmark and India studies cited above, or detrimental if there is an adverse additive or synergistic interaction with hepatitis B vaccination, which is also given shortly after birth in several nations. This might explain why a statistically significant difference in mean NMRs, IMRs, and U5MRs was found between nations giving no neonatal vaccine doses and those giving two doses, and why our 2019 and 2021 linear regression analyses show statistically significant positive correlations between the number of neonatal vaccine doses required by each nation and NMRs, IMRs and U5MRs.

Contribution of neonatal deaths to infant and under age five deaths

Approximately 45-75% of all infant deaths and 30-60% of under age five deaths occur during the neonatal period. About 75% of neonatal deaths occur during the first week of life [34], the same time period when the birth doses of required neonatal vaccines are administered. The worldwide contribution of neonatal deaths to the under age five mortality rate has increased from 37% in 1990 to 47% in 2019 [35,36]. The actual percentages differ by nation. For example, in 2021, Singapore's neonatal mortality rate comprised 43% of its IMR but just 35% of its U5MR. The US neonatal mortality rate comprised 61% of its IMR and 52% of its U5MR.

Younger age and lower weight

In a prior study [19], we investigated VAERS reports of hospitalizations and mortality among infants. Younger infants (who generally have lower weight) were significantly more likely than older infants to have had an adverse reaction to vaccines that required hospitalization (*r* = 0.97, *p* < .001). Vaccinated neonates had the highest hospitalization rate (as a proportion of total VAERS reports in their age group): 20.1% (95% CI: 17.3 to 23.0). Additionally, the mortality rate for vaccinated infants less than 6 months of age was significantly higher than that of vaccinated infants 6 months to 1 year of age: rate ratio, RR = 3.0 (95% CI: 2.6 to 3.4). 

Other studies using different methodologies provide additional evidence that younger age and lower weight increase the risk of serious adverse reactions to vaccines. Sen et al. [37] found that infants with major adverse reactions to vaccines were significantly younger and weighed less at the time of vaccination than infants who did not have major reactions. One-third (33.3%) of premature infants vaccinated at 70 days of age or less had major adverse reactions compared with none when vaccinated over 70 days of age. Mawson et al. [38] found that vaccinated infants born prematurely were five times more likely to be diagnosed with a neurodevelopmental disorder than vaccinated non-preterm children (OR 5.4, 95% CI: 2.5 to 11.9), and 14 times more likely when compared to children who were born full term and not vaccinated (OR 14.5, 95% CI: 5.4 to 38.7).

Misclassification of neonatal deaths

Complications of preterm birth are the main cause of neonatal deaths. Preemies have low birth weight and undeveloped immune systems yet neonatal vaccine dosages are not adjusted for weight. Studies provide evidence that premature newborns (and full-term babies with low birth weight) may be particularly susceptible to serious adverse reactions to vaccines, including life-threatening apnea. Sánchez et al. [39] found that 12% of vaccinated preterm infants experienced apnea within 72 hours post-vaccination. Preterm infants who were vaccinated at a lower weight had the most severe cases (*p* = 0.01). Apnea preceding sudden death has been well-documented [40,41].

DeMeo et al. [42] found that low birth weight infants were significantly more likely to develop sepsis, and require respiratory support and intubation in the three days post-vaccination compared to the three days prior to vaccination: RRs = 3.7, 2.1, and 1.7, respectively. Five infants died after receiving their vaccines. Recent data indicate that sepsis has an 18% mortality rate in neonates [43].

Pourcyrous et al. [44] found that when premature infants were given more than one vaccine concurrently, they were four times more likely to have adverse cardiorespiratory events (apnea, bradycardia, or oxygen desaturation) and abnormal C-reactive protein levels compared to preterm infants who received a single vaccine.

These studies indicate that some deaths attributed to preterm birth or low birth weight may be associated with the hepatitis B and/or BCG vaccines administered at birth, and the true cause of these deaths could be misclassified. In fact, misclassification of vaccine-related fatalities under alternate cause-of-death classifications may be unavoidable since cause-of-death classifications associated with infant vaccination do not exist. The official list of *130 Selected Causes of Infant Death* [45], compiled from the International Classification of Diseases (published by the World Health Organization) does not provide a medical code for vaccine-related mortality. Thus, doctors, coroners, and other medical examiners are effectively compelled to misclassify and conceal vaccine-related fatalities under alternate cause-of-death classifications. According to the CDC [46], "inaccurate or inconsistent cause-of-death determination and reporting hamper the ability to monitor national trends, ascertain risk factors, and design and evaluate programs to prevent these deaths."

Delayed neonatal fatalities

Vaccine doses (for hepatitis B and tuberculosis) given during the neonatal period are more highly correlated with IMRs (*r* = 0.46 in 2021) and U5MRs (*r* = 0.48) but less so with NMRs (*r* = 0.34). This suggests that some deaths associated with neonatal doses may be delayed. In a study of 30 future SIDS victims, Kahn et al. [40] found that obstructive and mixed apneas preceded their deaths by a median of 8 weeks. According to Albani et al., [47] "the findings in a certain number of 'near miss SIDS' cases support the hypothesis that there might be a link between some infants with prolonged sleep apnea and some later SIDS victims." Thus, some apneas that occur in the early neonatal period may not escalate to a sudden and complete expiration of life until weeks later, during the post-neonatal stage of infancy. If the apneas are induced by neonatal vaccines, this might explain why vaccine doses at birth correlate better with IMRs and U5MRs than NMRs.

Another hypothesis is that neonatal vaccines, through some priming mechanism [48] or cumulative toxicity may increase the risk of a severe or fatal reaction to subsequently administered vaccines. Vaccines have also been known to promote immune responses that can increase the risk of severe illness or death upon subsequent infection with the associated pathogen [49], although antibody-dependent enhancement (ADE) or vaccine-associated enhanced disease (VAED) is considered rare.

Sudden infant deaths

SIDS is the leading cause of death during the post-neonatal period [50]. Several studies provide evidence that a subset of infants may be especially susceptible to SIDS during the early post-vaccination period [51-53]. For example, Miller [51] found that infant deaths and SIDS cases were not randomly distributed each day. Instead, infant mortality and SIDS cases reported to VAERS tended to occur in temporal proximity to vaccine administration. Of 1048 SIDS cases, 51% clustered within 3 days post-vaccination and 76% within 7 days post-vaccination, a statistically significant finding (*p* < .00001).

Under age five vaccines

Based on 2019 and 2021 data, linear regression analyses of neonatal and infant vaccine doses versus U5MRs yield more robust positive correlations than under age five vaccine doses versus U5MRs. Our hypothesis is that the additional doses administered from the second through fifth years of life have less effect on under age five mortality than vaccines administered earlier in life. Toddlers that survived the neonatal and infancy periods may be less likely to have fatal reactions to vaccines. Children in the first year of life, as compared to the second through fifth years of life, weigh less and are receiving more vaccines in a shorter time period. Receipt of vaccines at a younger age (associated with lower weight), and multiple vaccines administered concurrently (as regularly occurs during infancy), have been shown to increase the risk of hospitalizations and death [19].

Effect of additional vaccine doses on mortality

In our 2021 analysis of the top 50 nations whereby vaccine doses range from 12 to 26, IMR increased by a factor of 0.167 deaths/1000 live births (95% CI: 0.0766 to 0.257) for each additional vaccine dose added to the immunization schedule (Table 6). Conversely, each reduction of six vaccine doses improves IMR by one death per 1000 live births.

Neonatal vaccine doses appear to have a significant impact on mortality. In 2021, there was a statistically significant difference of 1.28 (95% CI: 0.43-2.14) deaths per 1000 live births (*p* < .002) between the mean IMRs among nations that did not give their neonates any vaccine doses (mean IMR = 2.87) and those that required them to receive two vaccine doses (mean IMR = 4.15) (Table 5). Similarly, there was a statistically significant difference of 1.57 (95% CI: 0.58-2.57) deaths per 1000 live births (*p* < .001) between the mean U5MRs among nations that did not give their neonates any vaccine doses (mean U5MR = 3.43) and those that required them to receive two vaccine doses (mean U5MR = 5.00). Thus, developed nations that require their neonates to receive two vaccine doses would likely achieve a measurable improvement in childhood mortality rates simply by eliminating this requirement.

The statistically significant positive correlations between vaccine doses and mortality demonstrated in our analyses are plausible if an increase in all-cause mortality associated with some vaccines in developed nations is greater than the number of lives saved from potentially deadly infections specifically targeted by those vaccines. For example, neonatal vaccines designed to protect against hepatitis B and tuberculosis may not contribute to an overall reduction in mortality in nations whose infants are at low risk of mortality from these diseases. Vaccine policymakers have an obligation to determine the full impact of their current vaccination schedules on deaths from any cause. More safety research is needed on the number of childhood vaccines that are administered concurrently, cumulatively, and the sequence in which they are given [6,54], to confirm they are providing the intended effects on child survival.

Effect of additional nations in our current datasets

In our original 2009 linear regression analysis of vaccine doses routinely given to infants in developed nations and IMRs, just 29 nations had better IMRs than the US. By 2019, the US declined to 44th in IMR rank, and in 2021 the US ranked 50th in the world. When our 2019 linear regression analysis was limited to the top 20 nations (rather than the top 44), the correlation coefficient increased from *r* = 0.45 to *r* = 0.73 (*p* < .0003), revealing a strong direct relationship between the number of vaccine doses routinely administered during infancy and IMRs. Similarly, when our 2021 linear regression analysis was limited to the top 20 nations (rather than the top 50), the correlation coefficient increased from *r* = 0.47 to *r* = 0.62 (*p* < .004). As nations with higher IMRs are added to the dataset for analysis, the correlation coefficients incrementally decrease. This trend is as expected when a dataset of highly developed nations with relatively homogenous socioeconomic factors (that minimizes confounders) shifts to one with successively greater variability. A positive correlation between infant vaccines and IMRs becomes less detectable, or attenuated, in analyses of nations with greater heterogeneity of socioeconomic factors.

Strengths and limitations

Our prior linear regression analyses [7,8], and this present one, now comprise three years of data revealing statistically significant positive correlations between vaccine doses routinely given to infants in developed nations and IMRs: 2009 (*r* = 0.70, *p* < .0001); 2019 (*r* = 0.45; *p* < .002); and 2021 (*r* = 0.47; *p* < .0005). Herein, we also broadened our analyses to explore potential relationships between childhood vaccine doses and NMRs and U5MRs. Using 2019 and 2021 data, 17 of 18 analyses (12 linear regressions and six ANOVA and Tukey-Kramer tests) achieved statistical significance and corroborated the trend reported in our original study [7], demonstrating that as developed nations require more vaccine doses for their young children, mortality rates worsen.

Our datasets include the US, a nation that required the most vaccines for their infants, and all nations with better IMRs than the US. We did not analyze all nations since mixing highly developed and Third World nations would introduce confounding and bias arising from heterogeneity of socioeconomic factors and inconsistent vaccination rates (discussed in our previous paper [8]).

Vaccination rates in the US declined from 95% during the 2019-2020 school year (before the covid pandemic) to 94% during the 2020-2021 school year [55]. Many of the other nations in our 2021 dataset had similar small declines during the pandemic but still maintained relatively high vaccination rates (mainly exceeding 90%) [56]. Thus, it is unlikely that the pandemic caused an appreciable impact on the findings in our 2021 analyses. 

Due to the small number of nations in each group giving zero, one, or two neonatal vaccine doses, the statistical power is low in the one-way ANOVA and Tukey-Kramer tests conducted to determine differences in group mean NMRs, IMRs, and U5MRs.

Our analyses are ecological comparisons that provide insight into the possible relationship between exposure and outcome at a population level while the effect of any individual exposure-response is unknown. Analyses of the influence of vaccine doses on mortality rates could be improved if patient records were available detailing the date and type of each vaccine administered.

Consistent with our earlier studies of vaccine doses and mortality rates, we calculated the total number of vaccine doses given to children but did not differentiate between the substances, or quantities of those substances, in each dose. In addition, recommended vaccines that might have been administered to the newborn's mother during pregnancy, and their potential contribution to the total number of vaccine doses that the child receives (in utero and then after birth) were not considered.

## Conclusions

There are statistically significant positive correlations between neonatal, infant, and under age five mortality rates of developed nations and the number of early childhood vaccine doses that are routinely given. When developed nations require two versus zero neonatal vaccine doses, or many versus fewer infant vaccine doses, our study suggests there may be unintended consequences that increase all-cause mortality. Further investigations of the hypotheses generated by this study are recommended to confirm that current vaccination schedules are achieving their intended objectives.

## Appendices

Nations reporting six or fewer infant deaths were omitted from the main analyses due to IMR instability caused by excessively wide confidence intervals. This epidemiological convention recommended by the CDC and other health agencies was thoroughly discussed in our previous paper [8]. However, to avoid the perception of bias, the three nations listed in Table 7 for 2019, and four nations listed in Table 8 for 2021, have been added back into our datasets for reanalysis. The results are shown in Tables 9-12.

**Table 7:** 2019 data associated with the three nations excluded from the main analyses (due to small populations, few infant deaths, and IMR instability) NMRs: Neonatal Mortality Rates; IMRs: Infant Mortality Rates; U5MRs: Under Age 5 Mortality Rates

Nations	Doses <28d	Doses <1y	Doses <5y	NMRs <28d	IMRs <1y	U5MRs <5y
San Marino	0	23	31	0.86	1.62	1.85
Monaco	0	22	30	1.65	2.55	3.12
Andorra	0	19	36	1.51	2.50	2.95

**Table 8:** 2021 data associated with the four nations excluded from the main analyses (due to small populations, few infant deaths, and IMR instability) ^a^UNICEF datasets list an identical NMR and IMR for Monaco, possibly due to few raw deaths and IMR instability ^b^This nation requires a hepatitis B vaccine at birth NMRs: Neonatal Mortality Rates; IMRs: Infant Mortality Rates; U5MRs: Under Age 5 Mortality Rates

Nations	Doses <28d	Doses <1y	Doses <5y	NMRs <28d	IMRs <1y	U5MRs <5y
San Marino	0	23	31	0.80	1.53	1.72
Monaco	0	22	30	1.54^a^	1.54^a^	2.92
Andorra	0	19	36	1.39	2.64	2.76
Antigua & Barbados	1^b^	22	33	3.32	5.16	6.14

**Table 9:** Correlation coefficients (r) reported by linear regression analysis between the number of vaccine doses given during each childhood period and each mortality rate, 2019 and 2021 ^a^These values are not applicable (N/A) since many of the vaccine doses are given after the cutoff period. NMR: Neonatal Mortality Rate; IMR: Infant Mortality Rate; U5MR: Under Age 5 Mortality Rate

	2019 correlation coefficients (N = 47)			2021 correlation coefficients (N = 54)		
	NMR r (p-value)	IMR r (p-value)	U5MR r (p-value)	NMR r (p-value)	IMR r (p-value)	U5MR r (p-value)
Neonatal doses	0.35 (.016)	0.47 (.0008)	0.48 (.0006)	0.37 (.005)	0.49 (.0002)	0.50 (.0001)
Infant doses	N/A^a^	0.42 (.004)	0.39 (.007)	N/A^a^	0.43 (.001)	0.43 (.001)
U5 doses	N/A^a^	N/A^a^	0.30 (.042)	N/A^a^	N/A^a^	0.29 (.035)

**Table 10:** 2019 group mean mortality rates of nations requiring zero, one, or two neonatal vaccine doses, plus ANOVA and Tukey-Kramer p-values of differences between the group means ^a^All post hoc Tukey-Kramer tests for 2019 demonstrated a statistically significant (*p* < .05) pairwise difference between the mean mortality rates of nations requiring zero versus two neonatal doses. NMR: Neonatal Mortality Rate; IMR: Infant Mortality Rate; U5MR: Under Age 5 Mortality Rate

	2019 number of neonatal vaccine doses (N = 47)			p-value corresponding to...	
	Zero	One	Two	ANOVA	Tukey-Kramer^a^
NMR	1.95	2.16	2.70	.048	.038
IMR	2.78	3.23	4.10	.003	.002
U5MR	3.36	3.88	4.91	.002	.002

**Table 11:** 2021 group mean mortality rates of nations requiring zero, one, or two neonatal vaccine doses, plus ANOVA and Tukey-Kramer p-values of differences between the group means ^a^All post hoc Tukey-Kramer tests for 2021 demonstrated a statistically significant (*p* < .05) pairwise difference between the mean mortality rates of nations requiring zero versus two neonatal doses. NMR: Neonatal Mortality Rate; IMR: Infant Mortality Rate; U5MR: Under Age 5 Mortality Rate

	2021 number of neonatal vaccine doses (N = 54)			p-value corresponding to...	
	Zero	One	Two	ANOVA	Tukey-Kramer^a^
NMR	1.95	2.26	2.78	.02	.016
IMR	2.76	3.40	4.15	.001	.0007
U5MR	3.32	4.05	5.00	.0006	.0004

**Table 12:** Slopes of the best-fit line from each linear regression analysis of mortality rate versus the number of vaccine doses during each childhood period, 2019 and 2021 ^a^Slope represents the rate of change in deaths/1,000 live births per vaccine dose. NMR: Neonatal Mortality Rate; IMR: Infant Mortality Rate; U5MR: Under Age 5 Mortality Rate

Linear regression analysis	2019	2021
	Slope^a^ (95% confidence interval)	Slope^a^ (95% confidence interval)
NMR vs. neonatal doses	0.353 (0.068 – 0.638)	0.410 (0.126 – 0.694)
IMR vs. infant doses	0.135 (0.046 – 0.223)	0.162 (0.068 – 0.256)
U5MR vs. under age 5 doses	0.075 (0.003 – 0.146)	0.086 (0.006 – 0.166)

In summary, the differences between the findings in our main analyses and those reported in these supplementary tables are negligible. However, based on ANOVA tests using 2019 data, the group mean NMRs corresponding to groups of nations requiring zero, one, and two neonatal vaccine doses has now demonstrated a statistically significant difference between the group means. Thus, using 2019 and 2021 data, including nations with IMR instability, all 18 of 18 analyses (12 linear regressions and six ANOVA and Tukey-Kramer tests) achieved statistical significance and corroborated the positive associations previously reported in this paper.
